# The Relationship Between Serum Anti-Müllerian Hormone and Basal Antral Follicle Count in Infertile Women Under 35 Years: An Assessment of Ovarian Reserve

**DOI:** 10.7759/cureus.50181

**Published:** 2023-12-08

**Authors:** Ummey Nazmin Islam, Anwara Begum, Fatema Rahman, Md. Ahsanul Haq, Santosh Kumar, Kona Chowdhury, Susmita Sinha, Mainul Haque, Rahnuma Ahmad

**Affiliations:** 1 Obstetrics and Gynecology, Dhaka Medical College Hospital, Dhaka, BGD; 2 Obstetrics and Gynecology, Colonel Malek Medical College Hospital, Manikganj, Manikganj, BGD; 3 Bio-Statistics, Infectious Diseases Division, International Centre for Diarrhoeal Disease Research, Bangladesh (icddr, b), Dhaka, BGD; 4 Periodontology and Implantology, Karnavati School of Dentistry, Karnavati University, Gandhinagar, IND; 5 Pediatrics, Gonoshasthaya Samaj Vittik Medical College, Dhaka, BGD; 6 Physiology, Khulna City Medical College and Hospital, Khulna, BGD; 7 Research, School of Dentistry, Karnavati Scientific Research Center (KSRC) Karnavati University, Gandhinagar, IND; 8 Pharmacology and Therapeutics, National Defence University of Malaysia, Kuala Lumpur, MYS; 9 Physiology, Medical College for Women and Hospital, Dhaka, BGD

**Keywords:** bangladesh, > 35 years, bangladeshi female, infertility, diminished ovarian reserve, ovarian reserve, amh, anti mullerian hormone, afc, antral follicle count

## Abstract

Introduction

Estimating ovarian reserve has been the cornerstone of designing treatment plans for female infertility over the last few years. The most reliable biomarker for assessing female fertility is the antral follicle count (AFC). Also, the anti-müllerian hormone (AMH) is a sensitive test for predicting ovarian reserve and is precisely associated with AFC value.

Objective

The study aimed to investigate the relationship between serum AFC and AMH levels.

Methods

This cross-sectional type of observational study included 101 healthy infertile women aged 20-35 years and with low serum AMH. The mean difference in basal AFC among different age groups was evaluated using an independent sample t-test, revealing no significant difference. A multiple regression model was used to assess the association between serum AMH, and other factors related to demographics and other aspects of infertile women with basal AFC.

Results

The mean age of infertile women in our study was 30.7±3.69, and 29.7% of females had secondary infertility. The highest ovarian reserve was notable among the group 20-25 years, and the lowest follicular volume was observed in the 31 to below 35 years. Multiple regression analyses revealed that serum AFC and AMH had a strong positive association with basal ovarian volume. Additionally, every one-unit surge in AFC and AMH was statistically significant (p<0.05) and concomitant increases with 0.45 cc and 3.98 cc in basal ovarian volume, respectively.

Conclusion

The AMH and AFC strongly associate with basal ovarian volume, which declines as age progresses.

## Introduction

Globally, 8-10% of women have been reported as infertile [1]. There are multiple causes of infertility [2]; among those instigating factors, shrunken or meager ovarian reserve [3,4] is suggestive of substantial phasedowns in the ovarian enormity and follicle lagoon stockpile, consequently, come up with imperfect oocyte characteristic and childlessness [5-7]. Over the last few years, estimating ovarian reserve has been the cornerstone for designing the treatment plan for female infertility [8-12]. An ideal ovarian reserve test should be easy to execute, duplicated, noninvasive, and able to measure quantity accurately and, hence, the follicular pool's quality and predict the chance of pregnancy [13-16]. This concept has introduced various static and dynamic tests into clinical practice to evaluate ovarian reserve [17-19]. These include some static biochemical tests [13,20], such as baseline serum follicle stimulating hormone (FSH), baseline serum estradiol (E2), serum inhibin B, anti-müllerian hormone (AMH); some dynamic biochemical tests like clomiphene citrate challenge test, exogenous FSH ovarian reserve test (EFFORT), gonadotropin-releasing hormone agonist stimulation test (GAST) [13,18,21] and some biophysical examination - antral follicle count (AFC), ovarian volume and ovarian doppler [13,21-23]. Eventually, all of them except AFC and AMH are found to have limited value in clinical use [15,24-26].

AFC has been designated as the eminent precise biomarker to evaluate female reproductive physiology [27,28]. AFC is determined easily with the help of high-resolution sonographic systems [29,30]. Multiple studies reported that transvaginal ultrasound detected a constant decline in AFC with increasing age. This declining rate befitted considerably above 35 years among women possessing good health with normal menstrual physiology. Additionally, no evident cause of infertility [31-34]. Multiple studies reported that the number of AFCs was lower among infertile women than in fertile groups of similar age ranges and other necessary parameters [26,27,35-37]. One study reported the array of AFC in women exhibiting disorders of sterility was 5‑20, while that in women demonstrating natural reproductive function was 5‑17 [27]. Nevertheless, one more Indian study reported that the overall AFC among women (27.86±3.33 years) with the disorders of infertility was 3-24, whereas that in normal females (28.8±3.58 years) with confirmed typical reproductive function and pregnancy was 10-26 [36]. Another study in the USA comprises four age groups of 25-30, 31-35, 36-40, and 41-45 of both diagnosed infertile and fertile women who had statistically significantly (p<0.05) lower AFC among unfertile females, excepting 41-45 folk. The AFC range was 4-40, 1-30, 1-32, and 1-21 among infertile cases age assemblages of 25-30, 31-35, 36-40, and 41-45, respectively. Additionally, the AFC range was 4-58, 5-48, 0-52, and 1-33 among women with proven fertility age assemblages of 25-30, 31-35, 36-40, and 41-45, respectively [37]. An AFC less than 4 = meager count, 4-9 = low count, and concerned physicians guess about a likely or feasible to poor response to the stimulation. Furthermore, 9-13, 14-21, 22-35, and over 35 are considered somewhat reduced, intermediate, standard (good), and very high antral count, respectively [38]. There are multiple causes of low AFC, even among young females, such as genetic or chromosomal disorders, endometriosis, medical/surgical treatment, especially ovarian surgeries, immunological disorders, age, and lifestyle influences like smoking [39-41]. Among all factors, females over 35 remain the foremost cause of low AFC, resulting in infertility [26,41,42].

A girl child is born with 1-2 million primordial cells that characterize the oocyte's progenitor cells [26,43,44]. Nonetheless, females in their healthy lives suffer from a diminution of ovarian reserve, resulting in infertility and sub-fertility long before menopause, often because of diminished ovarian reserve [8,26,45,46]. AMH is synthesized by the ovary, explicitly growing small follicular granulosa cells [47-50]. AMH is also known as a Mullerian-inhibiting substance [51]. Additionally, AMH is a dimeric glycoprotein representing the transforming growth factor β (TGFβ) species and a robust predictive biomarker of ovarian reserve [22,52-54]. Blood serum AMH has been denoted as the consistent reference point in gauging reproductive aging (contrarily affiliated to the length of life). AMH reveals the persistent non-cyclic evolution of small follicles [55-57]. Thus, the AMH act is useful clinically, as this marker is considered more sensitive and predictive in detecting ovarian reserve or embryonic primordial follicular pool (the quantity of oocytes continues to exist in the ovaries) and is precisely associated with AFC value [15,22,42,47,55,58-60]. Multiple studies reported that serum AMH levels progressively fall off from age 25 years forward [61,62]. Various studies reported that AMH levels increased during juvenile and puberty [63-65], reaching the topmost level at 18 years [63]. After that, a declining phase started and continued throughout the reproductive age (19-50 years) [63]. Additionally, a Chinese study revealed that AMH levels started declining and drastically reduced by 30-34 and 45 years, among 28.1% and 79.4%, respectively [66,67]. Age is the robust dominant factor controlling female reproductive physiology, regardless of tribe, ethnicity, or race [67]. AMH signifies the amount of ovarian backup; nevertheless, it cannot assess the oocyte quality [68]. Multiple studies revealed that only AMH is not competent enough to appraise pregnancy outcomes in womenfolk [59,68,69]. Even then, AMH is a must biomarker test for therapeutic intervention among women seeking help in infertility clinics to evaluate ovarian reserve for over 30 years [20,49,50,69-71]. However, AMH possesses the strength to envisage the ovarian reciprocation to hyperstimulation during in vitro fertilization (IVF), to calibrate the time of menopause [49], and to specify iatrogenic impairment to the ovarian follicular pool [72]. One more research has reported that there is a nonexistence of a typical global level for AMH. This confines assessment between AMH analyses. Additionally, the intrinsic and extrinsic aspects that impact serum AMH quantity are unknown. After that, these issues restrict apposite clarification of AMH levels in a clinical scenario [47]. One study in Taiwan comprising 1935 childless womenfolk with a mean age of 35.1±4.7 years had a mean serum AMH level of 3.6 ± 2.8 ng/mL [73]. Another Taiwanese study had a population of 2,155. Among them, 972 and the rest 1183 were below 35 and greater than or equal to (≥) 35, respectively. This research reported that among women aged≥35 years, the AMH level was 2.9 ± 3.1 ng/mL [74]. Another study was conducted among 4556 childless females in China, Japan, Korea, Thailand, Vietnam, Malaysia, India, and Indonesia. This research found among age groups below 30, 30-31, 32-33, 34-35, 36-37, 38-39, and 40-40+years had AMH levels of 4.58±3.16; 4.23±3.23; 3.90±3.06; 3.21±2.65; 2.74±2.44; 2.30±1.91; 1.67±2.00 ng/ml, respectively. The overall AMH level among these multiethnic groups was 3.44 ± 2.93 ng/ml [75]. The median AMH values were 4.23 ng/mL, 3.48 ng/mL, 2.43 ng/mL, 1.28 ng/mL, and 0.52 ng/mL among Indian infertile females of age group in 20-25, 26-30, 31-35, 36-40 and 40-44 years, respectively [41]. This study also revealed that 55.7% of the infertile females below 35 years of age had low AMH; among these cases, 50.7% had low AFC correspondingly [75]. Another study conducted in the USA revealed that typically Caucasian women had higher levels than age-matched African and Hispanic females [67]. This study also reported that Chinese females below the age of 25 years had higher AMH levels than Caucasians [66]. One more study reported that the aging process is the principal determining factor for childlessness [76]. After that, AMH levels highly fluctuate between ethnic groups and races [66,67,77]. Additionally, serum AMH levels were presented to be arbitrarily vacillated right through the periodic cycle of women [78-81]. Furthermore, middling serum AMH intensities are considerably greater in the late follicular phase with ovulation than in the early luteal stage [82-84]. In contrast, multiple studies reported that AMH remains constant throughout the cycle (follicular, luteal stage, or any point) and even between menstrual periods. Consequently, an AMH test can be conducted during the menstrual cycle [85-88]. Several studies reported that reproductive and lifestyle features affect age-related AMH levels [89,90]. Multiple research projects revealed that prolonged use of birth-control hormonal agents affects AMH level [91-93]; nevertheless, AMH level returns to normal within 2-12 months of withdrawal of such pharmacological intervention [91,93].

Problem statement of infertility in Bangladesh

Infertility is a serious and sensitive health and social issue worldwide, affecting millions of people of reproductive age [94,95]. Childlessness is a distressing and painful incident for a couple [94], predominantly in developing women, including Bangladesh [96-99]. According to global data, almost one in six people experience infertility in their lifetime [100]. Bangladesh had no statistical evidence till 2012 regarding the prevalence rate of infertility [101]. A survey conducted in South Asian countries in Bangladesh regarding infertility in 2012 reported that the country had 4% infertile couples [101-103]. Furthermore, it has been reported that 18.9-36.8% of couple suffers from secondary infertility in Bangladesh [103,104]. It has been reported that Bangladeshi infertile couples, especially women, develop psychiatric disorders and increase healthcare overhead of community and out-of-pocket expenses individually [105]. Additionally, Bangladesh is an overpopulated country. Therefore, the primary emphasis of health policy is population control [106,107]. After that, infertility is a ramshackle or run-down health distress in Bangladesh [101]. Although a few studies regarding infertility have been conducted among Bangladeshi people, not much work has been directed at accessing ovarian reserve and its two determining factors: AFC and AMH.

Infertility and sub-fertility are widespread ailments affecting an estimated 50-70 million populace internationally [108,109]. The World Health Organization (WHO) appraised that 9% of couples globally mêlée with fertility disorders and considered a “social disease” [109] and that the male factor contributes to 50% of infertility disorders [108,109].

Objectives of the study

The study aimed to examine the association between two promising tests for checking ovarian reserve that are performed nowadays: serum AFC and AMH levels in younger, apparently healthy infertile women with diminished ovarian reserve to plan an appropriate strategy at the earliest stage of infertility management. This study also examined the association between AFC and factors like age and AMH with basal ovarian volume.

## Materials and methods

Study design

**Figure 1:**
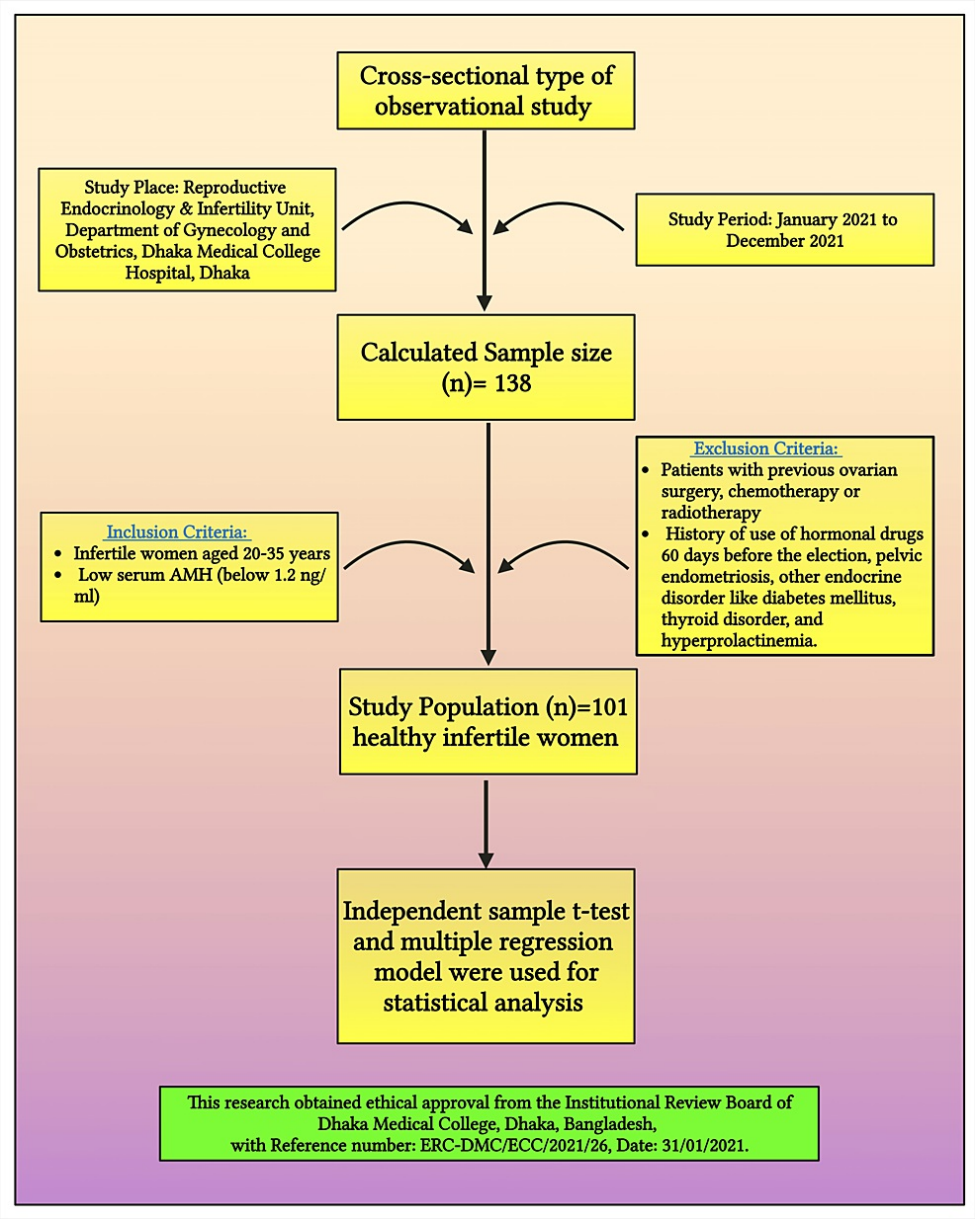
This figure illustrates the methodology of the study. Notes: This figure has been drawn with the premium version of BioRender (https://biorender.com; accessed on 16 November 2023) with the license number QR263OOXJ6. Image credit: Susmita Sinha AMH: anti-Müllerian hormone

It was a cross-sectional observational study (Figure 1). The research was conducted in the Reproductive Endocrinology and Infertility Unit, Department of Gynecology and Obstetrics, Dhaka Medical College Hospital, Dhaka, Bangladesh, and included healthy infertile women below 35 years of age with diminished ovarian reserve. A purposive sampling method was employed which took place from January 2021 to December 2021. The following formula was applied to calculate the sample size, according to this formula: n = z^2^pq/d^2^ where n is the required sample size, Z=1.96 at a 95% confidence interval, P is the prevalence of the condition (10% or 0.1); Q = 1-p = (1-0.1) = 0.9, and d (Degree of accuracy) is 5% or 0.05 (it means the accepted standard error limit considered 5% in this study). Substituting these values, n = z^2^pq/d^2^ = [(1.96)^2^ X 0.1 X 0.9]/(0.05)^2^ = 138. Consequently, the targeted sample size was n = 138. However, due to the COVID-19 pandemic, a targeted sample size could not be achieved. This research had given its best effort, and was able to find 101 subjects for the current study.

Research subjects

Inclusion Criteria

Infertile women aged 20-35 years and low serum AMH (below 1.2 ng/ml).

Exclusion Criteria

Patients with previous ovarian surgery, chemotherapy or radiotherapy, history of use of hormonal drugs 60 days before the election, pelvic endometriosis, and other endocrine disorders like diabetes mellitus, thyroid disorder, and hyperprolactinemia.

Statistical analysis plan

The data in the analysis table includes mean values with standard deviations for continuous variables and numbers with percentages for categorical variables. The mean difference in basal AFC among different age groups was evaluated using an independent sample t-test. A multiple regression model was used to assess the association between serum AMH, and other factors related to demographic and other aspects of infertile women with basal AFC, including all the elements in the same model. The statistical software STATA-15 (StataCorp LLC, College Station, Texas, USA) was used for the statistical comparisons, and graphical presentations were created using GraphPad Prism 8.3.2 (GraphPad Software, San Diego, CA). A p-value of 0.05 was considered statistically significant.

Ethical approval

This research obtained ethical approval from the Institutional Review Board of Dhaka Medical College, Dhaka, Bangladesh, with reference number: ERC-DMC/ECC/2021/26, dated 31/01/2021.

## Results

Table 1 provides an analysis of the essential characteristics of the study participants. The table includes observations based on a total of 101 individuals. The mean age of the participants is 30.7 years, with a standard deviation of 3.69. The age distribution shows that the largest age group is 26-30 years, which accounts for 72.3% of the participants. Regarding religion, most participants are Muslim, comprising 93.1% of the total, while Hindus make up 6.93%.

**Table 1:** Basic characteristics of the study participants Notes: Data was presented as mean±SD or number with percent in the parenthesis or medium with minimum or maximum.

Variables	Observation (n=101)
Age	30.7±3.69
Age group	
20-25 years	8(7.92%)
26-30 years	73(72.3%)
31-<35 years	20(19.8%)
Religion	
Muslim	94(93.1%)
Hindu	6(6.93%)
Occupation	
Housewife	78(77.2%)
Service	23(22.8%)
Education qualification	
Illiterate	2(1.98%)
SSC or below	39(38.6%)
HSC	12(11.9%)
Graduate	12(11.9%)
Master’s or above	36(35.6%)
Locality	
Slum	9(8.91%)
Urban	58(57.4%)
Rural	34(33.7%)
Income category	
Low	30(29.7%)
Medium	64(63.4%)
High	7(6.93%)
Duration of infertility	
1-5 years	59(58.4%)
6-10 years	31(30.7%)
>10 years	11(10.0%)
Type of infertility	
Primary	71(70.3%)
Secondary	30(29.7%)
Menstruation cycle	
<21 days	2(1.98%)
21-35 days	76(75.3%)
>35 days	23(22.8%)
Menstruation duration, median (min, max)	4(1, 12)
Menstrual flow	
Average	72(71.3%)
Scanty	18(17.8%)
Heavy	11(10.9%)
History of abortion	
Yes	21(20.8%)
No	80(79.2%)
Body mass index (BMI), Kg/m^2^	
Underweight	5(4.95%)
Normal	33(32.7%)
Overweight	51(50.5%)
Obese	12(11.9%)

Regarding occupation, most participants (77.2%) are housewives, while 22.8% are engaged in service occupations. The education qualifications of the participants vary, with 1.98% being illiterate, 38.6% having SSC or below education, 11.9% having HSC education, 11.9% being graduates, and 35.6% having a master’s degree or above.

The participants were from diverse localities, with 8.91% residing in slum areas, 57.4% in urban areas, and 33.7% in rural areas. Regarding income, 29.7% of participants fall into the low-income category, 63.4% in the medium-income category, and 6.93% in the high-income category.

The duration of infertility varies among the participants, with 58.4% experiencing infertility for 1-5 years, 30.7% for 6-10 years, and 10.0% for more than 10 years. Most participants (70.3%) have primary infertility, while 29.7% have secondary infertility.

The table also provides information on the participants' menstrual cycle, duration of menstruation, menstruation flow, history of abortion, and body mass index (BMI) categories. However, the table does not provide specific numbers or percentages for the menstrual cycle variables.

**Figure 2:**
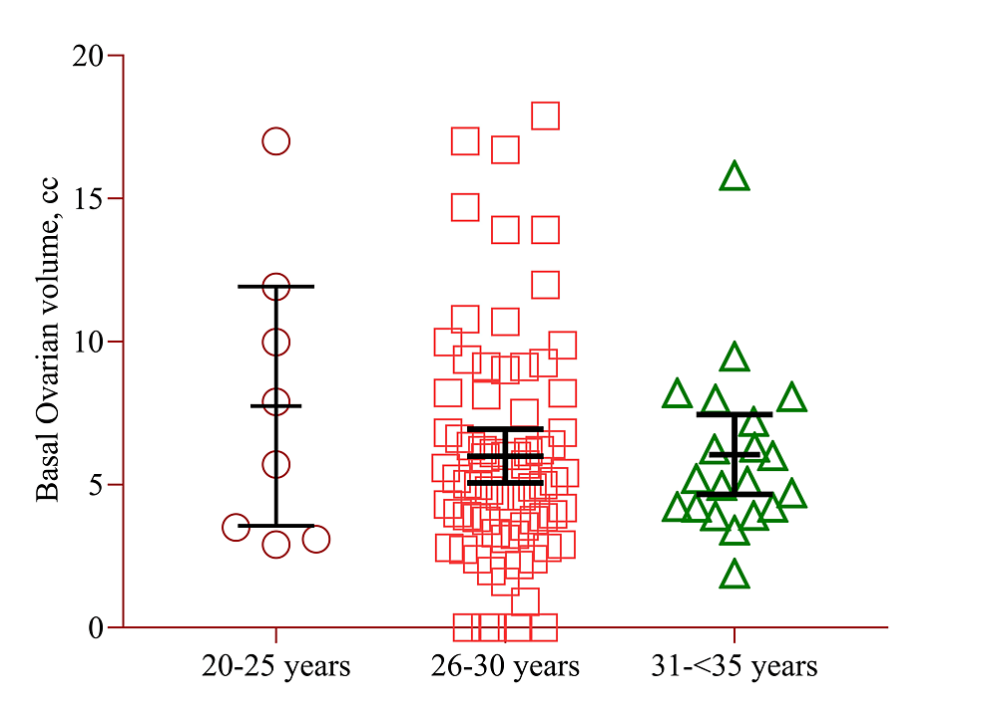
Mean difference of basal antral follicle count with age group. Notes: Data has been presented as mean±SD. Independent sample t-test was used to check the significance level.

Basal ovarian volume showed a decline with age. The highest volume was noted in the 20-25 years age group (7.75±5.00), and the lowest volume was observed in the 31-<35 years age group (6.01±3.93) (Figure 2). However, they declined and did not show any statistically significant difference.

Table 2 presents a multiple regression analysis examining the association between AFC and other associated factors with basal ovarian volume. This research rigorously examined the association between serum AFC and AMH with basal ovarian volume, treating both AFC and AMH as exposures and basal ovarian volume as the outcome variable. Our statistical approach involved the utilization of a multiple regression model that incorporated all relevant exposure variables. Every one-unit enhancement in (AFC and AMH) is associated with 0.45 cc (95% CI=0.21, 0.68, p<0.001) and 3.98 cc (95% CI=1.84, 6.12, p<0.001) increase in basal ovarian volume. It was also observed that an increase in age was associated with 0.27 cc (95% CI=0.02, 0.51, p=0.036) enlarged basal ovarian volume every year.

**Table 2:** Association of AFC and other associated factors with basal ovarian volume Notes: The multiple regression model was used by including all the principal exposures and associated factors in the same regression model to estimate the p-value. AMH: anti-Müllerian hormone, AFC: antral follicle count

	Basal ovarian volume	
	β Coefficient (95% CI)	p-value
AFC	0.45(0.21, 0.68)	<0.001
Serum AMH	3.98(1.84, 6.12)	<0.001
Age, in years	0.27(0.02, 0.51)	0.036
Income category		
Low	Ref.	
Medium	-0.48(-2.17, 1.20)	0.572
High	1.52(-4.72, 1.67)	0.346
Locality		
Urban	Ref.	
Slum	-2.03(-4.89, -0.02)	0.037
Rural	0.02(-1.60, 1.65)	0.979
Duration of infertility		
1-5 years	Ref.	
6-10 years	-0.16(-1.81, 1.49)	0.849
>10 years	1.14(-1.45, 3.72)	0.385
Menstruation flow		
Heavy	Ref.	
Average	-0.26(-2.74, 2.23)	0.838
Scanty	-1.35(-4.39, 1.69)	0.377
History of abortion		
No	Ref	
Yes	-0.69(-2.49, 1.12)	0.452
Body mass index (BMI), Kg/m^2^		
Normal	Ref.	
Underweight	0.74(-2.88, 4.36)	0.686
Overweight	-0.46(-2.14, 1.21)	0.584
Obese	0.94(-1.50, 3.38)	0.447

## Discussion

WHO defines infertility as “a disease of the male or female reproductive system defined by the failure to achieve a pregnancy after 12 months or more of regular unprotected sexual intercourse" [97]. Childlessness is a medical ailment [110] that often instigates emotional, bodily, cerebral, religious, saintly, increased stress, depression, anxiety, and medical impairments among the sufferer; female partners remain considerably more likely to face these agonies and pain [111-117]. The inimitable characteristic of this medical disease enmeshed distressing both the case and the patient's connubial spouse as a couple with martial frustration; again, women are the principal victims [116,117]. Nevertheless, contemporary data on infertility internationally are missing [118]; it has been projected that 48 million husbands and wives [118] and another 186 million people around the globe breathe with childlessness [118-120]. Multiple studies revealed that 8-12% of families are suffering from infertility and childlessness [121-123], and the rate of infertility fluctuates from country to country [120,123]; especially in high-income and developed countries, the infertility rate is declining than the rest of the globe [124].

**Figure 3:**
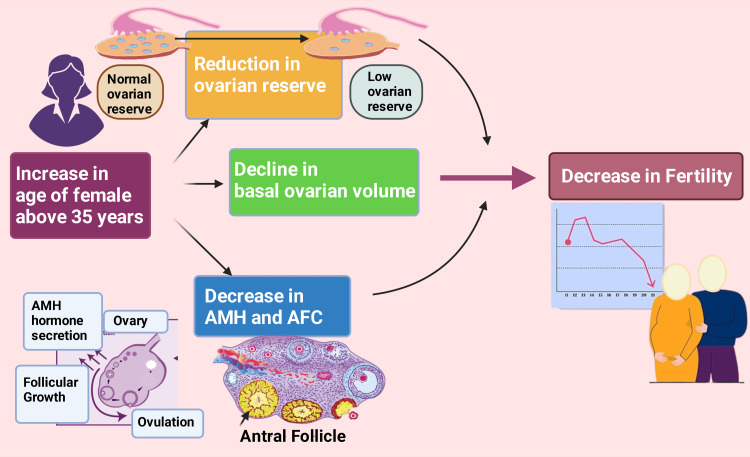
The figure demonstrates the effects of aging on female fertility. Notes: This figure has been drawn with the premium version of BioRender (https://biorender.com/; accessed on 8 November 2023) with license number OY262L8AFZ. Image credit: Rahnuma Ahmad AMH: anti-Müllerian Hormone, AFC: antral follicular count

The natural aging process in women decreases or causes a loss of number and features her oocyte and follicular lagoon. This process is considered a physiological outcome of menstrual cycle non-uniformity and the eventual termination of periodical bleeding [44,125-128]. After that, women beyond 35 years old and onward have their attempts to conceive delayed (Figure 3) [129-131]. Relatively older females’ granulosa cells produce AMH and inhibin B to a lesser extent than younger females because of decreased oocyte and follicular pool [130,132,133]. Multiple studies reported that those women who have infertility concurrently had psychological stress, e.g., anxiety, depression, psychological trauma [113,134-137]. Additionally, several research reported that poor nutritional status of women promotes infertility [138-140].

The mean age of infertile women in our study was 30.7±3.69, and around 30% of females had secondary infertility. Basal ovarian volume decreased as the aging process progressed. Again, the highest ovarian reserve was notable among the group 20-25 years, and the lowest follicular volume was observed in the 31 to below 35 years. Multiple regression analyses revealed that serum AFC and AMH had a strong positive association with basal ovarian volume. Additionally, every one-unit surge in AFC and AMH was statistically significant (p>0.05) and concomitant increases with 0.45 cc and 3.98 cc in basal ovarian volume, respectively.

The mean age of infertile women in our study was 30.7±3.69. Amraei et al. reported from Iran the mean age of infertile females was 29.6±5.5 [141]. One Indian study said that 39.3%, 12.6%, and 13.6% of infertile women in Central India are 25-29, 20-24, and 30-34 years, respectively [96]. In Nepal, the average age of infertile women was 27.44±5.214 years attending the infertility clinic of a tertiary hospital [142]. Another Nepalese study reported that the majority (51.52%) of infertility females were aged 26-30 [143]. One more study from Nepal revealed females attending infertility clinic preponderance (56%) aged 26-35 years [144]. The range and mean±SD age of the females was 16-46, 32.4±7.4 years among patients attending the infertility clinic of a Teaching Hospital in Sudan [144]. After that, our findings were almost like earlier studies around the globe. Our study found around 30% of females had secondary infertility. Nevertheless, one Indian study revealed that approximately 6% of females had secondary infertility [145]. Another critical finding in India is that infertility varies from 2.5 to 13.9% from region to region [138]. In contradiction, another conducted among Indian infertile females revealed that 42.5% were suffering from secondary infertility [146]. Multiple Indian research showed about 28.6-53% of females had complained of secondary infertility, especially those women who got married over the age of 30 years [123,147]. Multiple studies reported that secondary infertility was found to be higher than primary childlessness [148], and the range was found to be 32.63-34.1% in low and middle-income countries (LMICs) [149,150]. The findings regarding secondary infertility were in the same line of studies conducted by various LMICs.

This current study found that basal ovarian volume decreased as the aging progressed. Multiple studies reported a similar biological continual physiological event [126,151-153]. Our analysis detected that the highest ovarian reserve was notable among the group 20-25 years, and the lowest follicular volume was observed in the 31 to below 35 years. Cleveland Clinic, USA, reported that greater levels of AMH interpret more eggs and a better ovarian pool [154]. Additionally, multiple studies said AMH levels come into existence all the while of pubescence and reach the highest point around 25 years old [154,155]. It has been further reported that among females with good physical condition, the ovarian lagoon is determined by Graafian follicular span of life, and antral follicular age was contemplated as identical to their length of life [132,156]. After that, the earlier findings were the same as the current study. Gunasheela et al. reported that ovarian reserve declines below age 35 [41]. One more study said that the typical follicular quantity springs up from 0.7 ml (95% CI 0.4-1.1 mL) at two years of age to reach its highest point of 7.7 ml (95% CI 6.5-9.2 ml) at the age of 20; then started to decline to nearby 2.8 ml (95% CI 2.7-2.9 ml) at the cessation of periodical menstrual cycle and after that, more diminishes follicular pool [157]. Wallace and Kelsey reported that the females' ovarian reservoir was statistically significantly smaller among those above 35 than those below 35 years of age [158]. Thus, our study findings were almost like earlier studies.

**Figure 4:**
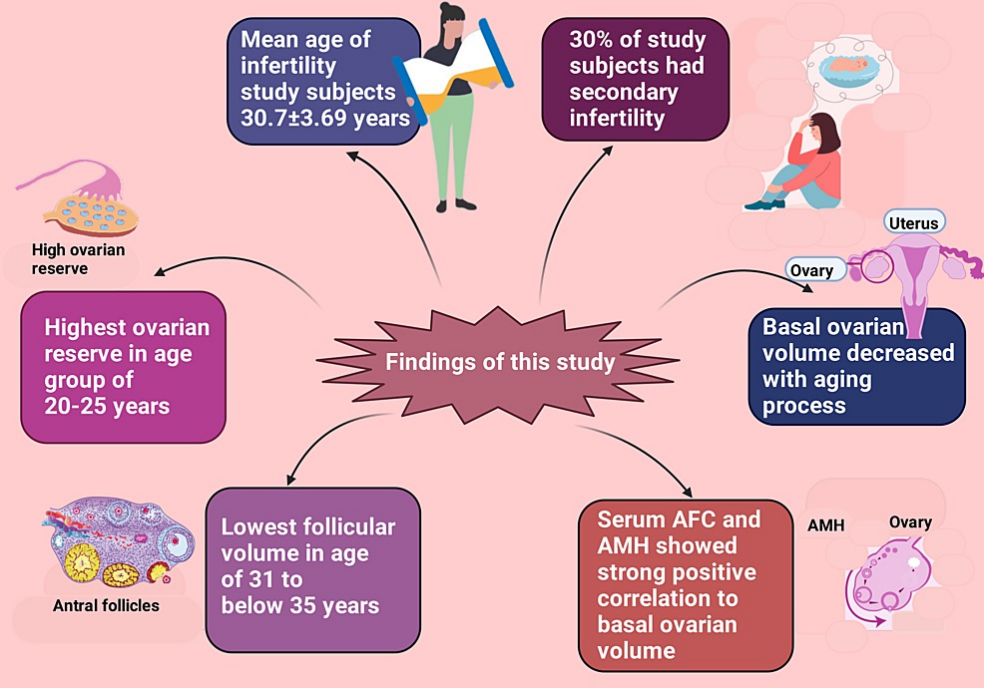
The figure illustrates the results obtained in this study. Notes: This figure has been drawn with the premium version of BioRender (https://biorender.com/; accessed on 8 November 2023) with license number OY262L8AFZ. Image credit: Rahnuma Ahmad AMH: anti-Müllerian hormone, AFC: antral follicular count

This study conducted multiple regression analyses and revealed that serum AFC and AMH had a strong positive association with basal ovarian volume. Additionally, we detected that every one-unit surge in AFC and AMH were statistically significant (p>0.05) concomitant increases with 0.45 cc and 3.98 cc in basal ovarian volume, respectively. It has been reported that there is a statistically significant fall in ovarian volume every 10 years of females' lives from 30 to 70. Moreover, the average ovarian lagoon in pre-menopausal females is significantly superior to post-menopausal women folk [159]. Nevertheless, one earlier study revealed that ovarian reserve started declining after the age of 20 years [157]. American College of Obstetricians and Gynecologists reported that female reproductive physiology and reproduction remain at their peak from late adolescence/young adulthood (18 to 21 years) to late 20s (among 27-29 years old) [160]. Additionally, when females reach 30 years and more, chances to conceive naturally fall [160-162]. Furthermore, there is deterioration of reproductive physiology by the age of 35-36 [160,163]. Moreover, for those women aged ≥40 to 45 years, her ability to get pregnant without assisted reproductive techniques (ART) remains most improbable [160,164-166]. The mean age of our study population was 30.7±3.69 years (Figure 4). Earlier findings were from separate ethnic and socio-cultural groups; therefore, our results were somewhat different.

Limitations of the study

A smaller sample size and single-center may influence the outcomes, while a larger sample size with nationwide data can enhance both statistical power and the strength of the statistical evidence.

## Conclusions

Female fertility remains closely associated with age, as found in the current study and earlier research conducted around the globe. The ovarian volume reduces with age, and secondary infertility is higher in incidence. However, this decline in fertility principally determines patients’ age and ovarian, and follicular lagoon may vary in different ethnic groups. The AMH and AFC also strongly correlate with basal ovarian volume and decline with age. The onset of secondary infertility related to age, in most cases, has been reported in female to be in their 30s. Physicians and other health care providers may disseminate the knowledge of the significance of age for reproductive health and successful conception in females.

Studies must be carried out on large-scale populations to understand the mechanisms linking the aging process to ovarian and reproductive health. The current study findings may help develop pharmacological and other lifestyle interventions that may slow down or even reverse the effects of aging on female reproduction. Additionally, the current research results could be a springboard for future research. Also, policies may be designed to spread awareness regarding the adverse effects of age on reproduction so that women may be able to make an informed decision when they wish to conceive. Healthy lifestyle adoption needs to be promoted amongst the population, which may positively impact the people and help them have a successful reproductive life.
